# A multicrystal diffraction data-collection approach for studying structural dynamics with millisecond temporal resolution

**DOI:** 10.1107/S2052252516016304

**Published:** 2016-10-26

**Authors:** Robin Schubert, Svetlana Kapis, Yannig Gicquel, Gleb Bourenkov, Thomas R. Schneider, Michael Heymann, Christian Betzel, Markus Perbandt

**Affiliations:** a University of Hamburg c/o DESY, Notkestrasse 85, 22603 Hamburg, Germany; b The Hamburg Center for Ultrafast Imaging c/o DESY, Luruper Chaussee 149, 22761 Hamburg, Germany; c Center for Free Electron Laser Science c/o DESY, Notkestrasse 85, 22607 Hamburg, Germany; d EMBL c/o DESY, Notkestrasse 85, 22603 Hamburg, Germany

**Keywords:** time-resolved crystallography, fixed target, multicrystal data collection, room temperature, synchrotron radiation, radiation damage, structure determination, protein structure, X-ray crystallography, structural biology

## Abstract

Many biochemical processes take place on timescales ranging from femto­seconds to seconds. Accordingly, any time-resolved experiment must be matched to the speed of the structural changes of interest. Therefore, the timescale of interest defines the requirements of the X-ray source, instrumentation and data-collection strategy. In this study, a minimalistic approach for *in situ* crystallization is presented that requires only a few microlitres of sample solution containing a few hundred crystals. It is demonstrated that complete diffraction data sets, merged from multiple crystals, can be recorded within only a few minutes of beamtime and allow high-resolution structural information of high quality to be obtained with a temporal resolution of 40 ms. Global and site-specific radiation damage can be avoided by limiting the maximal dose per crystal to 400 kGy. Moreover, analysis of the data collected at higher doses allows the time-resolved observation of site-specific radiation damage. Therefore, our approach is well suited to observe structural changes and possibly enzymatic reactions in the low-millisecond regime.

## Abstract 2

*In situ* crystallization using a Kapton sandwich assembly allows diffraction data to be recorded from multiple protein crystals at room temperature with millisecond temporal resolution at high-brilliance synchrotron X-ray radiation sources.

## 1. Introduction   

X-ray crystallography remains the most extensively used method to determine the three-dimensional structure of biological macromolecules and has supported the understanding of the chemical mechanisms underlying biological function in recent decades. During the last few years, the development of dedicated micro-crystallography at synchrotron-radiation (SR) sources and X-ray free-electron lasers (XFELs) has revolutionized the field. Today, high-resolution diffraction data can be obtained from microcrystals and nanocrystals as long as the crystallites are well ordered (Riekel *et al.*, 2005 ▸; Moukhametzianov *et al.*, 2008 ▸; Chapman *et al.*, 2011 ▸; Boutet *et al.*, 2012 ▸; Redecke *et al.*, 2013 ▸; Smith *et al.*, 2012 ▸; Neutze & Moffat, 2012 ▸; Spence *et al.*, 2012 ▸; Weckert, 2015 ▸; Gruner & Lattman, 2015 ▸). Although sample handling has been automated at many microfocus beamlines by using crystal-mounting robots, mechanical stress can be introduced to crystals by the transfer from the growth environment as well as by buffer and temperature changes. Sample handling is especially difficult for fragile crystals and therefore several methods have been proposed to minimize the extent of manual handling during this procedure (Cipriani *et al.*, 2012 ▸). Although diffraction data collection has predominantly been performed at cryogenic temperatures, in some cases cryocooling can hinder straightforward diffraction data collection at SR beamlines. Identifying the optimal composition of a cryoprotectant can be cumbersome and may have a detrimental effect on the quality of the crystal and its diffraction properties (Axford *et al.*, 2012 ▸). Furthermore, freezing can diminish conformational diversity, and different conformational distributions can even be observed at cryogenic temperatures compared with room temperature (Rasmussen *et al.*, 1992 ▸; Tilton *et al.*, 1992 ▸; Fraser *et al.*, 2009 ▸, 2011 ▸; Juers & Matthews, 2004 ▸). New data-collection strategies at room temperature can minimize these unintended effects and can additionally pave the way for kinetic crystallography to follow the biological reactions of proteins in a crystalline lattice. However, in order to address this, new sample-mounting systems for *in situ* crystallography (McPherson, 2000 ▸) such as low X-ray-absorbing 96-well plates (Kisselman *et al.*, 2011 ▸; Axford *et al.*, 2012 ▸), microfluidic chips (Pinker *et al.*, 2013 ▸; Guha *et al.*, 2012 ▸; Perry *et al.*, 2013 ▸; Heymann *et al.*, 2014 ▸), capillaries (Stellato *et al.*, 2014 ▸) and other fixed targets (Coquelle *et al.*, 2015 ▸; Huang *et al.*, 2015 ▸; Feld *et al.*, 2015 ▸; Mueller *et al.*, 2015 ▸) have been developed in order to record diffraction data from protein microcrystals at room temperature. For serial femtosecond crystallography (SFX) at free-electron laser radiation sources liquid-jet technology has been developed, which has the advantage of rapidly delivering microcrystals in suspension to the X-ray beam (Redecke *et al.*, 2013 ▸; Schlichting, 2015 ▸; Martin-Garcia, 2016 ▸). However, the presently relatively low hit rates as well as the high sample consumption limit the applicability of this sample-delivery approach. To reduce sample consumption, liquid-jet technology has been extended to operate with lipidic cubic phase (LCP; Weierstall *et al.*, 2014 ▸) and other high-viscosity sample-delivery media (Conrad *et al.*, 2015 ▸).

In contrast to liquid jets, which usually have a diameter of 5 µm or lower, viscous jets are much harder to focus and typically jet diameters of 25 µm or larger are used. Therefore, they result in an increased overall background-to-noise ratio, with additional background scattering from the media used to increase viscosity (Conrad *et al.*, 2015 ▸).

The success of serial femtosecond crystallography at FELs has catalyzed experimental approaches aiming to perform serial crystallography with microcrystals at the more prevalent and readily accessible SR sources (Gati *et al.*, 2014 ▸; Stellato *et al.*, 2014 ▸; Botha *et al.*, 2015 ▸; Nogly *et al.*, 2015 ▸; Zander *et al.*, 2015 ▸; Roedig *et al.*, 2015 ▸, 2016 ▸).

Radiation damage can be either classified as specific or global and occurs at room temperature, which limits the number of diffraction patterns that can be obtained from a single crystal. Typically, data are collected at room temperature using a large number of crystals to spread the total dose over the ensemble. At an optimized SR source beamline with a flux of approximately 5 × 10^12^ photons per second at 12 keV, focused to match the size of a microcrystal, the tolerated dose of each crystal at room temperature is limited to exposure times of a few milliseconds to avoid significant crystal damage. Subsequently, data from a succession of microcrystal exposures are merged to acquire complete data sets. However, the success of the methods applied so far for room-temperature data collection using microcrystals is limited either by a rather high consumption of crystal suspensions or by the crystal quality, which often suffers from introduced mechanical stress. Some of these latest diffraction data-collection methods also have to deal with the problem of increased background scattering, and thus suffer from a reduced signal-to-noise ratio (Panneels *et al.*, 2015 ▸; Liu *et al.*, 2013 ▸).

We have designed and established a minimalistic fixed-target approach and a corresponding data-collection protocol that can be easily adapted at appropriate microfocus synchrotron beamlines. In addition, the protocol optimized and applied in this study requires minimal crystal manipulation prior to data collection and eliminates the need for cryoprotectants, which might reduce diffraction quality. In order to test and verify this approach, we have performed room-temperature data collection using high-brilliance synchrotron radiation at PETRA III from multiple crystals to investigate specific and global radiation-damage effects in the millisecond regime. To address this question and approach, thaumatin from *Thaumatococcus daniellii* containing eight intramolecular disulfide bonds was used as a target, because it is a standard protein that has already been well characterized in radiation-damage studies (Garman, 2010 ▸). The analysis of diffraction data collected using this new method showed a dose-dependent destabilization of the disulfide bonds present in thaumatin, temporally resolved in the millisecond regime.

## 2. Materials and methods   

### 2.1. Sample preparation and crystallization   

Lyophilized thaumatin (from *T. daniellii*; Sigma–Aldrich catalogue No. T7638) was used without further purification. A protein solution at a concentration of 34 mg ml^−1^ was prepared by dissolving the protein in a buffer consisting of 50 m*M* bis-tris pH 6.5. The protein solution was centrifuged at 20°C for 15 min at 16 100*g* before use. The final protein concentration was verified photometrically using a NanoDrop system (Thermo Scientific) using an extinction coefficient of 29 420 calculated using *ProtParam* (Gasteiger *et al.*, 2005 ▸). Supersaturation of the protein solution was induced by the addition of a precipitant solution consisting of 1.3 *M* sodium tartrate, 50 m*M* Tris pH 6.8, followed by thorough mixing. All solutions were prepared using ultrapure water and were filtered through a 0.2 µm filter (Sartorius Stedim).

### 2.2. Set-up of the fixed-target Kapton sandwich   

Thaumatin crystals were obtained by adding 2 µl reservoir solution to 2 µl protein solution in a modified hanging-drop vapour-diffusion setup on a Kapton foil of 8 µm thickness (American Durafilm) covered by a cover slide on a pre-greased Linbro plate. To facilitate assembly, a small drop of water was placed on the glass lid to aid mounting of the foil. Since both the Kapton foil and the glass slide are hydrophilic, the water droplet pulls the foil and slide together through capillary force. Since the mounting droplet evaporates over a few hours, separation of the slide and foil was trivial after crystallization. We observed crystals to grow to a final size of 50–100 µm in diameter, usually after 1 d. Upon lifting the cover slide, excess grease was removed and a second Kapton foil was gently placed to seal the crystal-containing drop, resulting in a thin crystal suspension layer between the Kapton foils. The Kapton-foil sandwich was sealed with grease, which prevents the sample suspension from drying out. The sandwich was fixed using double-sided adhesive tape on a frame (1 × 1 cm or in SBS format) to be mounted on a kappa goniostat or a plate goniometer, respectively. Both frame types were produced in-house using a table-top three-dimensional printer (Ultimaker 2 from Ultimaker BV or Form 1 from Formlabs Inc.).

### 2.3. Data collection, scaling and refinement   

Diffraction data were collected on EMBL beamline P14 at the DESY storage ring PETRA III in Hamburg, Germany using a beam size of 10 × 5 µm (FWHM of Gaussian profile) at 296 K. X-rays with an energy of 12.8 keV and a flux of 2.2 × 10^12^ photons s^−1^ in a non-attenuated beam were used and diffraction patterns were recorded using a PILATUS 6M hybrid pixel detector. A total of 60 thaumatin crystals were exposed to X-rays and 20 diffraction patterns with a total oscillation-angle range of 20° were recorded from each crystal within 800 ms in shutterless operation. The exposure time per image of 40 ms was limited by the maximal frame rate of the detector. Two separate data collections were performed from different sets of crystals in order to determine the maximum tolerated X-ray dose without radiation damage and to further analyze the time-resolved propagation of specific radiation damage. For the first data-collection run a transmission of 50% (1.1 × 10^12^ photons s^−1^) was used, while the transmission in the second run was reduced to 5% (1.1 × 10^11^ photons s^−1^).

Each single diffraction pattern of thaumatin was individually processed using *XDS* (Kabsch, 2010 ▸). For each time slice (frame) individual HKL files from all crystals were created and scaled using *XSCALE*. The correlation coefficients between data sets from the individual crystals were greater than 90%, which indicates a high degree of isomorphism. In order to determine the highest resolution shell, the conservative criterion 〈*I*/σ(*I*)〉 (>2) was used. The X-ray dose applied to each crystal at different time intervals was calculated using *RADDOSE* (Zeldin *et al.*, 2013 ▸).

The phases for model building were obtained by molecular replacement using *MOLREP* (Vagin & Teplyakov, 2010 ▸) from the *CCP*4 suite (Winn *et al.*, 2011 ▸) and using the three-dimensional coordinates for thaumatin from Protein Data Bank (PDB) entry 1lr2 as a search model (Charron *et al.*, 2002 ▸). All structures were refined isotopically using *REFMAC*5 (Winn *et al.*, 2011 ▸; Murshudov *et al.*, 2011 ▸), and *Coot* (Emsley *et al.*, 2010 ▸) was used for visual inspection of the final model. Solvent molecules were automatically added during the refinement process and checked to confirm that they were at chemically reasonable positions, at which difference electron density also exceeded the 3σ level. All models were inspected for Ramachandran outliers. The coordinates for the structures, as well as the experimental diffraction amplitudes, have been deposited in the PDB (http://www.rcsb.org) as entries 5lh0, 5lh1 and 5ln0 for the low-dose run, and 5lh3, 5lh5, 5lmh, 5lh6 and 5lh7 for the high-dose run.

### 2.4. Decay of diffraction power   

To follow the decay of diffraction power over time, as described by Owen *et al.* (2014 ▸), the total sum of *I*/σ(*I*) for all indexed reflections on each recorded diffraction image, given by *XDS* (Kabsch, 2010 ▸), was taken as a reference value for every exposed crystal. The diffraction power of each crystal was normalized to the mean diffraction power of the first recorded image. By plotting the decay in diffraction power over time, a statistical distribution of the decay was observed.

### 2.5. Crystal orientations   

The distribution of the crystal lattice orientations with respect to the laboratory coordinate system was evaluated by determining the Euler angles from the *XDS* orientation matrix given in the output file XPARM.XDS (Kabsch, 1988 ▸) using *MATLAB* (release 2007a, The MathWorks). A detailed description of the calculation has been published by Zarrine-Afsar *et al.* (2012 ▸). The resulting Euler angles for the three rotation planes *xy*, *xz* and *zy* were grouped in classes of 10° and plotted as a histogram.

### 2.6. Detection of site-specific radiation damage   

Structure-factor amplitude Fourier difference maps *F*_o_ − *F*_o_ between different time intervals of data sets from thaumatin were calculated as described by Coquelle *et al.* (2015 ▸). The refined models from data collected within two different time intervals were superimposed using *PHENIX* (Adams *et al.*, 2010 ▸). Difference maps from different time intervals were then calculated using *Coot* (Emsley *et al.*, 2010 ▸). The difference density maps (*F*_o_^frame *x*^ − *F*_o_^frame *y*^) were inspected at a contour level of 4σ to identify differences.

## 3. Results and discussion   

### 3.1. On-foil vapour-phase crystallization and Kapton-foil sandwich   

**Figure 1:**
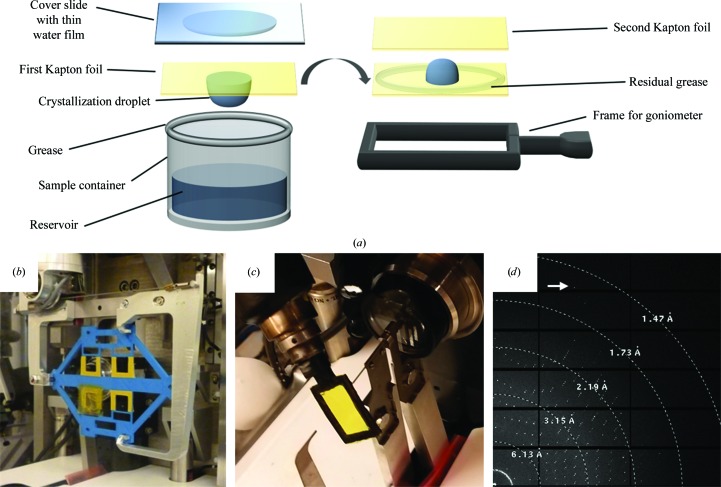
Crystallization setup and mounting of the Kapton-foil sandwich at the beamline. (*a*) Schematic representation of the hanging-drop vapour-diffusion experiment on Kapton foil and its fixation on a frame using double-sided adhesive tape. Individual Kapton sandwiches can be mounted on (*b*) a plate goniometer or (*c*) a goniometer with kappa geometry. (*d*) Diffraction data of thaumatin crystals in the Kapton sandwich were recorded to a resolution of 1.6 Å with a negligibly low background.

The aim of this study was to establish a setup and a protocol for X-ray diffraction data collection at room temperature, providing millisecond temporal resolution. Particular care was taken to design a reliable system that was as simple as possible, easy to fabricate, reproducible, and compatible with adaptation to standard goniometers. To achieve this, while also minimizing the extent of crystal manipulation, the protein crystals were grown on a Kapton foil in a hanging-drop approach. Once *in situ* crystallization has been successful, the crystal suspension can be directly sealed with a second Kapton foil prior mounting this Kapton-foil sandwich onto the goniometer (Figs. 1 ▸ *a*, 1 ▸ *b* and 1 ▸ *c*). Exposed crystals of thaumatin diffracted to a resolution of 1.6 Å (Fig. 1 ▸ *d*). It was observed that the X-ray background contribution of the thin Kapton double layer is rather low and is mostly limited to polymer scattering rings at 33 Å (2θ ≃ 1.7°) and 11 Å (2θ ≃ 5°) at a wavelength of 0.97 Å, not disturbing the data processing.

### 3.2. Data-quality and diffraction-intensity decay   

Diffraction data sets were collected by exposing thaumatin crystals in two separate experiments at low and at high X-ray photon fluxes of 1.1 × 10^11^ photons s^−1^ (low-dose experiment) and 1.1 × 10^12^ photons s^−1^ (high-dose experiment), respectively. To study possible radiation-damage effects, 20 consecutive exposures were recorded from a single crystal in both the low-dose and the high-dose experiment. Diffraction data from identical time intervals were indexed and merged from 46 crystals, resulting in 20 complete data sets collected at 20 time intervals, covering an exposure-time range of 800 ms for both the high-dose run and the low-dose run. The statistics of selected data sets at different time intervals are presented in Table 1 ▸. The total doses for the high-dose and low-dose runs after recording 20 consecutive diffraction patterns were calculated to be 2.32 MGy (2.9 MGy s^−1^) and 0.23 MGy (0.29 MGy s^−1^), respectively.

For the low-dose data only a minor decrease in the integrated high-resolution Bragg reflection intensities was observed. The maximum resolution decreased from 1.88 Å for the first data set (0–40 ms; total average dose of 0.01 MGy) to 1.96 Å for the last data set (760–800 ms; total average dose of 0.23 MGy). The data statistics demonstrate that reliable and complete diffraction data sets without significant global radiation damage have been recorded at each time interval.

**Figure 2:**
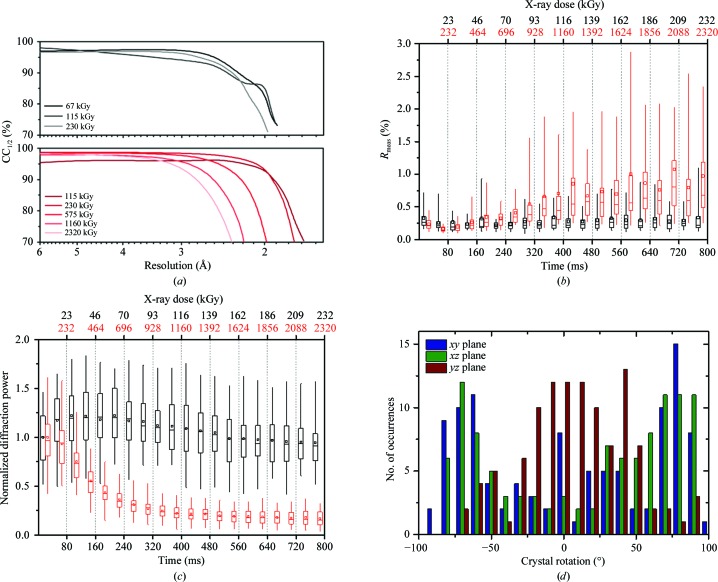
Statistics from room-temperature data collection from thaumatin crystals in the Kapton-foil sandwich. (*a*) CC_1/2_ values of the recorded diffraction data for the low-dose (black) and high-dose (red) experiments are plotted as a function of resolution. (*b*) Evolution of the *R*_meas_ value over time in the low-dose (black boxes) and high-dose (red boxes) experiments. (*c*) Intensity decay of thaumatin crystals as a function of time in the low-dose (black boxes) and high-dose (red boxes) experiments. The box plots in (*b*) and (*c*) represent the decay of diffraction intensities and *R*_meas_ of all exposed crystals (*n* = 46). The box represents the spread of 50% of all values, which are separated into the upper and lower quartiles by a horizontal band (median); the mean value is indicated by a small rectangle. Whiskers (vertical lines above and below the box) indicate the spread of 95% of all values. (*d*) Distribution of thaumatin crystal orientations in the Kapton-foil sandwich with respect to the laboratory coordinate system. The bipyramidal thaumatin crystals showed a broad distribution of orientations covering nearly 180° in the *xy* (blue), *xz* (green) and *yz* (red) planes.

In contrast, for the high-dose experiment comparison of the first (0–40 ms; total average dose of 0.12 MGy) with the last (760–800 ms; total average dose of 2.32 MGy) data set revealed that the maximum resolution decreased from 1.65 to 2.28 Å, indicating significant global radiation damage (Table 1 ▸, Fig. 2 ▸ *a*). Accordingly, the CC_1/2_ value also fell below 90% at lower resolution for data sets subjected to a total average dose of more than 500 kGy (Fig. 2 ▸ *a*). The data for the low-dose run showed no significant variation in the distribution of *R*_meas_ values over time for individual crystals. The mean *R*_meas_ values were persistently below 25% (Fig. 2 ▸ *b*). However, the data sets in the high-dose run showed a significant time-dependent increase in the mean *R*_meas_ value. In particular, the variation over all determined *R*_meas_ values for individual crystals became substantially larger with increasing total X-ray dose.

The intensity decay of the normalized diffraction power over time for the high-dose and low-dose experiments is shown in Fig. 2 ▸(*c*). The diffraction power in the high-dose run had already started to decrease after the first exposure and was below 50% after recording approximately four images (160 ms exposure time; ∼460 kGy dose). In contrast, when using a tenfold attenuated beam in the low-dose experiment, the diffraction power remained nearly stable over an 800 ms exposure time. This is in good agreement with the expected maximum dose tolerance of 430 kGy for a single thaumatin crystal at room temperature (Leal *et al.*, 2013 ▸), and is also higher than the commonly assumed dose tolerance of 300 kGy for other protein crystals at room temperature (Owen *et al.*, 2006 ▸; Nave & Garman, 2005 ▸).

However, all refined models at the selected time intervals presented in Table 1 ▸ reveal inconspicuous *R* factors/*R*_free_ values, with constant *R* values below 20%. In general, no increase in the refinement *R* values is observed with respect to the X-ray dose absorbed by the crystals. The final electron-density maps were of very good quality and all models have good stereochemistry.

### 3.3. Crystal orientations   

In previous diffraction data-collection approaches using X-ray-transparent chips, the orientation and arrangement of the crystals have been deliberately manipulated in order to obtain a random distribution of crystal orientations. This is owing to the fact that crystals will mostly settle onto a crystal facet when transferred onto a grid for diffraction experiments. To prevent this, the hydrophobicity and roughness of a silicone mesh chip covered with polyimide film was increased by adding small glass beads (Zarrine-Afsar *et al.*, 2012 ▸).

In the present study, no additional material was introduced. Therefore, we investigated the unit-cell orientation of all exposed crystals with respect to the laboratory coordinate system and demonstrated that a broad distribution of crystal orientations is obtained, even without selective manipulation (Fig. 2 ▸ *d*). For the bipyramidal thaumatin crystals no preferred orientations were observed in the *xy* plane, while the crystal orientations in the *xy* and *yz* planes are not completely random. This could be owing to crystals detaching and re­orienting during the sandwich assembly or even assuming a partially preferred orientation during crystal growth. However, the broad range of crystal rotations results in a sufficiently good coverage of reciprocal space as well as in complete data sets. Thus, no care needs to be taken when selecting crystals for X-ray exposure.

### 3.4. Time-resolved changes in the electron-density map   

**Figure 3:**
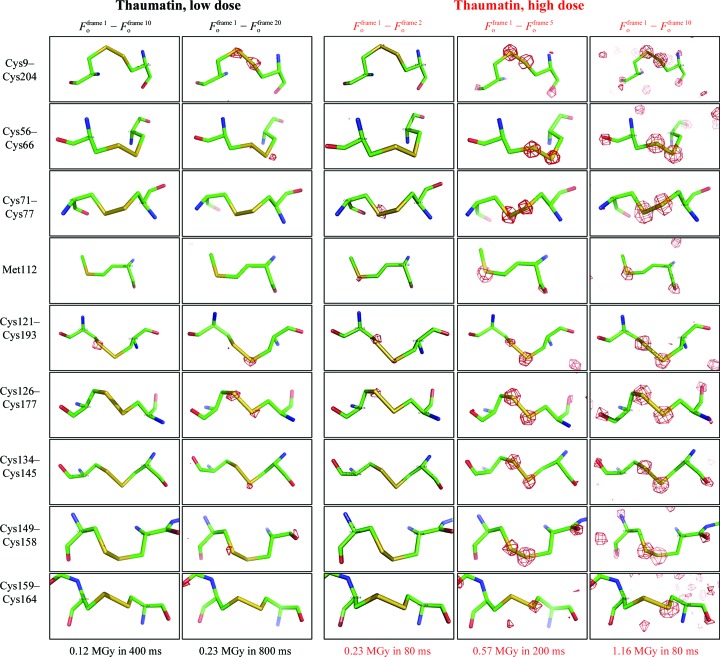
Time-resolved observation of specific radiation damage around all S atoms of thaumatin over time. Structure-factor amplitude Fourier difference maps *F*_o_ − *F*_o_ were calculated between different time intervals of X-ray exposure for the low-dose (left side, black) and high-dose (right side, red) experiments. The maps are displayed with red contours at 4σ indicating negative electron density.

The disulfide bridges of thaumatin are known to be sensitive to radiation damage (Garman, 2010 ▸; Yorke *et al.*, 2014 ▸). To visualize the temporal progression of the specific radiation damage, structure-factor amplitude Fourier difference maps *F*_o_ − *F*_o_ have been calculated between data sets for the first recorded diffraction pattern and those at corresponding later time intervals. The temporal resolution in our experiment was limited to 40 ms, based on to the maximal frame rate of the detector. However, our experiment can potentially easily be combined with the additional use of Hadamard transform-based X-ray probe–pulse sequences (Yorke *et al.*, 2014 ▸). Thereby, the temporal resolution for tracking biological processes may be further improved drastically to the low-microsecond regime. The data statistics indicate that strong radiation damage occurred in the high-dose diffraction data sets, while only minor radiation damage occurred in the low-dose experiment. The site-specific component of the radiation damage becomes visible by monitoring the difference density contoured at ±4σ in the proximity of all thaumatin S atoms (Fig. 3 ▸). Site-specific damage was prominently observed for the S atoms and minor damage was observed for the O atoms of some carboxyl groups.

As expected from the small decay of the diffraction intensity in the low-dose run, no specific radiation damage was observed for the data set collected in the time interval between 360 and 400 ms (∼0.12 MGy total average dose). Even for the data set collected in the time interval between 760 and 800 ms (∼0.23 MGy total average dose), only minor difference density could be detected around some of the disulfide bridges. This shows that the bonds between cysteines are still intact and presumably only start to become destabilized. This observation holds also true for data collected in the high-dose run within the 40–80 ms exposure time interval, with the same total absorbed average dose of ∼0.23 MGy. In contrast, more significant site-specific damage could already be observed for the data set collected within 160–200 ms exposure time (∼0.57 MGy total average dose) in the high-dose experiment (Fig. 3 ▸). All of the eight disulfide bonds reveal significant radiation damage. In contrast to our results, it was very recently reported that no indications of site-specific radiation damage up to the same absorbed dose of 0.57 MGy were observed for insulin (Roedig *et al.*, 2016 ▸). Roedig and coworkers concluded that specific radiation damage, and here in particular cleavage of disulfide bridges, is less temperature-dependent than global radiation damage and generally occurs only at higher doses. They assumed further that disulfide-bond breakage was not the preferred damage pathway at room temperature, where global radiation damage to the lattice was clearly the dominating effect. However, our data on thaumatin crystals do not support this general hypothesis. The sensitivity for specific radiation damage also depends on the sample.

## 4. Conclusion   

In this study, we have demonstrated that high-quality diffraction data sets with a temporal resolution of 40 ms can be recorded at room temperature by merging data collected from fewer than 50 protein crystals. The sample-preparation and data-collection strategy is straightforward. Using an atten­uated X-ray beam, 20 diffraction data sets over a total X-ray exposure period of 800 ms could be recorded with no significant site-specific or global radiation damage, if a maximum dose tolerance up to 400 kGy is considered. At doses higher than 550 kGy, beside the expected global radiation damage, we were able to observe dose-dependent site-specific damage most prominently at the radiation-sensitive disulfide bonds. The temporal resolution of 40 ms could be further reduced to less than 2 ms by using a non-attenuated X-ray beam in combination with the latest-generation EIGER 4M pixel detector, where diffraction patterns can be recorded at 750 Hz.

## Supplementary Material

PDB reference: thaumatin, low dose, 5lh0

PDB reference: 5lh1

PDB reference: 5ln0

PDB reference: high dose, 5lh3

PDB reference: 5lh5

PDB reference: 5lmh

PDB reference: 5lh6

PDB reference: 5lh7

## Figures and Tables

**Table 1:** Data-collection and refinement statistics for thaumatin using high-dose and low-dose X-ray photon fluxes at different time intervals Values in parentheses are for the highest resolution shell.

	Low-dose exposure			High-dose exposure				
	Frame 1 (0–40 ms)	Frame 10 (360–400 ms)	Frame 20 (760–800 ms)	Frame 1 (0–40 ms)	Frame 2 (40–80 ms)	Frame 5 (160–200 ms)	Frame 10 (360–400 ms)	Frame 20 (760–800 ms)
Data-collection statistics								
Beamline	P14	P14	P14	P14	P14	P14	P14	P14
Wavelength (Å)	0.96863	0.96863	0.96863	0.96863	0.96863	0.96863	0.96863	0.96863
Space group	*P*4_1_2_1_2	*P*4_1_2_1_2	*P*4_1_2_1_2	*P*4_1_2_1_2	*P*4_1_2_1_2	*P*4_1_2_1_2	*P*4_1_2_1_2	*P*4_1_2_1_2
Unit-cell parameters (Å)								
*a* = *b*	58.44	58.43	58.45	58.43	58.42	58.42	58.49	58.45
*c*	151.58	151.53	151.59	151.58	151.59	151.59	151.77	151.62
No. of crystals	46	46	46	46	46	46	46	46
Resolution (Å)	30–1.88 (1.95–1.88)	30–1.90 (1.97–1.90)	30–1.96 (2.02–1.95)	30–1.65 (1.71–1.65)	30–1.69 (1.75–1.69)	30–1.96 (2.03–1.96)	30–2.15 (2.23–2.15)	30–2.28 (2.36–2.28)
Total average dose (MGy)	0.01	0.12	0.23	0.12	0.23	0.57	1.16	2.32
Temperature (K)	296	296	296	296	296	296	296	296
*R*_p.i.m._ †	9.0 (30.6)	8.8 (31.0)	8.3 (31.9)	8.2 (33.2)	6.8 (43.5)	8.5 (39.5)	10.2 (46.6)	11.6 (49.3)
Measured reflections	62822	63464	54468	94713	90316	59357	41592	32153
Unique reflections	19955	19881	17759	29947	28198	18364	13192	10726
Average *I*/σ(*I*)	5.3 (2.0)	6.3 (2.1)	6.0 (2.0)	5.6 (2.1)	7.1 (2.1)	5.8 (1.9)	6.1 (2.1)	5.9 (2.0)
Mn(*I*) half-set correlation CC_1/2_	97.9 (71.5)	99.0 (78.2)	98.7 (72.9)	97.3 (70.7)	99.0 (71.2)	98.6 (65.0)	98.3 (66.9)	97.9 (61.7)
Completeness (%)	92.6 (93.6)	92.5 (93.0)	91.5 (92.8)	92.0 (92.0)	92.9 (93.8)	93.1 (93.4)	91.1 (90.6)	90.1 (90.1)
Multiplicity	3.15	3.19	3.07	3.16	3.20	3.2	3.15	3.00
Refinement statistics								
Resolution range (Å)	30–1.88	30–1.90	30–1.96	30–1.65	30–1.69	30–1.96	30–2.15	30–2.28
*R*/*R*_free_ (%)	18.8/23.9	18.1/22.8	18.2/22.4	19.3/22.9	17.6/20.1	17.6/22.0	17.0/23.6	17.2/23.2
Protein atoms	1550	1550	1550	1550	1550	1550	1550	1550
Water molecules	51	44	72	64	68	71	62	46
Ligand molecules	20	20	20	20	20	20	20	20
R.m.s. deviations								
Bond lengths (Å)	0.020	0.021	0.015	0.025	0.025	0.015	0.019	0.019
Bond angles (°)	2.04	2.12	1.72	2.29	2.63	1.68	2.08	2.07
*B* factors (Å^2^)								
Protein	22.6	25.0	27.1	22.3	25.1	29.6	31.1	30.6
Water	23.2	24.8	32.1	25.9	21.0	50.2	35.2	34.3
Ligand	20.4	47.1	47.2	34.1	43.3	34.5	91.8	115.74
Ramachandran plot analysis (%)								
Most favoured regions	97.67	99.51	97.07	98.53	98.53	97.07	97.56	97.07
Allowed regions	2.44	0.49	2.44	1.47	1.47	2.44	2.44	2.93
Generously allowed regions	0.49	0.00	0.49	0.00	0.00	0.49	0.00	0.00

† *R*_p.i.m._ = 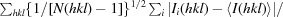 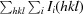, where 〈*I*(*hkl*)〉 is the mean intensity of the reflections *hkl*, 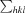 is the sum over all reflections and 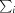 is the sum over *i* measurements of reflection *hkl*.
